# Conduit margin heating and deformation during the AD 1886 basaltic Plinian eruption at Tarawera volcano, New Zealand

**DOI:** 10.1007/s00445-016-1006-7

**Published:** 2016-02-13

**Authors:** Jenny Schauroth, Fabian B. Wadsworth, Ben Kennedy, Felix W. von Aulock, Yan Lavallée, David E. Damby, Jérémie Vasseur, Bettina Scheu, Donald B. Dingwell

**Affiliations:** Department of Earth and Environmental Science, Ludwig-Maximilians-Universität (LMU), Theresienstr. 41, 80333 Munich, Germany; Department of Geology, University of Canterbury, Private Bag 4800, Christchurch, New Zealand; School of Earth, Ocean and Ecological Science, University of Liverpool, Brownlow Street, Liverpool, UK

**Keywords:** Pyroclastic dyke, Volcanic conduit, Magma–rock interaction, Strain localisation, Wall rock heating

## Abstract

During explosive eruptions, a suspension of gas and pyroclasts rises rapidly within a conduit. Here, we have analysed textures preserved in the walls of a pyroclastic feeder dyke of the AD 1886 Tarawera basaltic Plinian fissure eruption. The samples examined consist of basaltic ash and scoria plastered onto a conduit wall of a coherent rhyolite dome and a welded rhyolitic dome breccia. We examine the textural evidence for the response of the wall material, built of ∼75 vol.% glass and ∼25 vol.% crystals (pore-free equivalent), to mass movement in the adjacent conduit. In the rhyolitic wall material, we quantify the orientation and aspect ratio of biotite crystals as strain markers of simple shear deformation, and interpret juxtaposed regions of vesiculation and vesicle collapse as evidence of conduit wall heating. Systematic changes occur close to the margin: (1) porosity is highly variable, with areas locally vesiculated or densified, (2) biotite crystals are oriented with their long axis parallel to the margin, (3) the biotites have greater aspect ratios close to the margin and (4) the biotite crystals are fractured. We interpret the biotite phenocryst deformation to result from crystal fracture, rotation and cleavage-parallel bookcase translation. These textural observations are inferred to indicate mechanical coupling between the hot gas-ash jet and the conduit wall and reheating of wall rock rhyolite. We couple these observations with a simple 1D conductive heating model to show what minimum temperature the conduit wall needs to reach in order to achieve a temperature above the glass transition throughout the texturally-defined deformed zone. We propose that conduit wall heating and resulting deformation influences conduit margin outgassing and may enhance the intensity of such large basaltic eruptions.

**Electronic supplementary material:**

The online version of this article (doi:10.1007/s00445-016-1006-7) contains supplementary material, which is available to authorized users.

## Introduction

Records of explosive basaltic eruptions of Plinian intensities are improving (e.g., Thordarson and Self 2003; Sable et al. 2009; Costantini et al. 2010; Marzoano et al., 2013) and although some authors have proposed eruption mechanisms (e.g., Houghton et al. 2004; Houghton and Gonnermann 2008; Sable et al. 2009; Costantini et al. 2010; Goepfert and Gardner 2010; Kennedy et al. 2010), the mechanics remain debated. Explanations for the exceptional explosivity of basaltic magmas include magma-water interaction (e.g., Houghton et al. 2004), high magma viscosities due to degassing-induced microlite crystallisation or due to cooling of the magma triggered by degassing (Houghton and Gonnermann 2008; Sable et al. 2009; Goepfert and Gardner 2010), critical bubble volumes causing the magma to disrupt (Goepfert and Gardner 2010), high degrees of outgassing contributing to higher melt viscosities and lower ascent rates (Houghton et al. 2004), instantaneous drops in the external pressure triggering high decompression rates (Costantini et al. 2010) and a decrease in conduit wall porosity leading to conduit wall sealing (Kennedy et al. 2010). Here, we explore the conduit wall sealing hypothesis further and analyse the textural record of such a process in order to evaluate if it is one possible contributing factor to high explosivity events at basaltic volcanoes.

The most powerful known examples of basaltic Plinian eruptions include the ∼60-ka Fontana Lapilli and the ∼2-ka Masaya Triple Layer deposits in Nicaragua, the 122 BC eruption of Etna and the AD 1886 Tarawera eruption in New Zealand (e.g., Houghton et al. 2004; Costantini et al. 2010). A recent example of such intense basaltic behaviour is the 2011 eruption of Grímsvötn in Iceland (e.g., Marzano et al. 2013). The basaltic dykes that feed these eruptions are rarely exposed. The AD 1886 Tarawera fissure eruption, however, is known to have exposed at least 16 basaltic feeder dykes (Nairn and Cole 1981). According to Nairn and Cole (1981), the dykes represent segments of the vent system where the eruption did not become highly explosive. Nevertheless, they provide an opportunity to examine the interaction between pyroclasts and conduit wall rocks during eruption.

Conduit wall properties may influence the behaviour of ascending magma and eruption dynamics (e.g., Stasiuk et al. 1996; Houghton et al. 2004; Rust et al. 2004; Kennedy et al. 2010). Conduit wall porosity is highly variable (Rust et al. 2004; Kennedy et al. 2010) due to interchangeable contribution from fractures, vesicles and cavities. The loss of gas from ascending magma can occur through such permeable structures (Jaupart 1998), reducing gas pressure and thus regulating the explosivity of eruptions (Stasiuk et al. 1996; Castro et al. 2012a, 2014). The presence of glass in the groundmass of conduit wall rocks will modify the wall rock behaviour on heating. Upon sufficient reheating, the glass will soften at the glass transition temperature interval, above which it is a supercooled viscous liquid. Hence, magma can exhibit both brittle and viscous deformation upon eruption (Dingwell and Webb 1989, 1990), healing of fractures (Tuffen et al. 2003; Cordonnier et al. 2012), vesiculation (e.g., Proussevitch and Sahagian 1996) and pore collapse (e.g., Westrich and Eichelberger 1994). As the rheology of bubbly magmas exhibits dependence on bubble content, capillary numbers and magma ascent rates (Manga et al. 1998; Llewellin and Manga 2005), along with temperatures and coupled stresses from the conduit interior, a change in bulk porosity can also lead to varying strain rates (e.g., Russell and Quane 2005) in the wall rock. As a result, the mechanical response (brittle vs. ductile) of the conduit lining to magma motion in the conduit is dependent on the magmatic overpressure triggering tensile failure (e.g., Heiken et al. 1988), and on the conduit wall porosity influencing the tensile strength. This has implications for the eruption style (Rust et al. 2004) and may influence parameters such as the threshold and depth of fragmentation (Kennedy et al. 2005).

Kennedy et al. (2010) postulated, based on experiments, that conduit wall softening via heating by the magma and potential decrease in wall rock permeability eventually yields a closed degassing system on the timescale of the 1886 Tarawera eruption. Here, we examine and discuss caveats to this conclusion with reference to the high along-fissure variability in eruption style during the AD 1886 event at Tarawera, while providing evidence that reheating occurred in the conduit margin and viscous shearing of conduit wall rock from interaction with gas-pyroclastic jets.

## Background: dykes feeding the AD 1886 tarawera volcano eruption

**Fig. 1:**
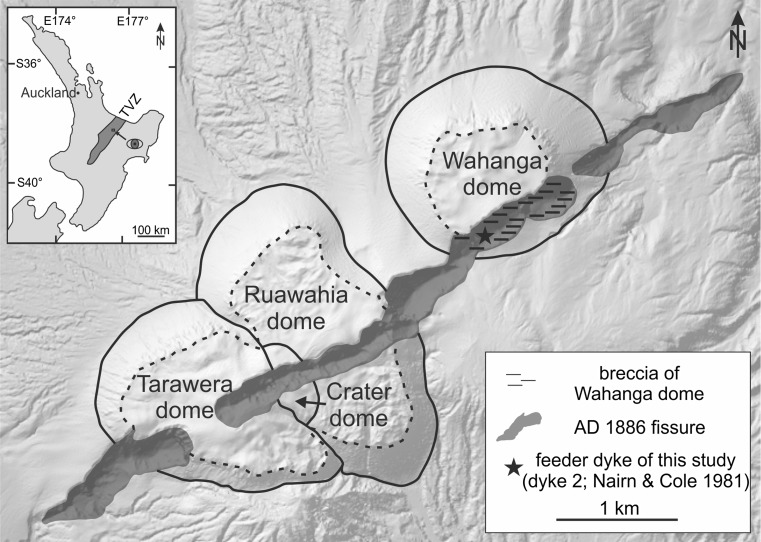
A digital elevation model showing the position and orientation of the AD 1886 basaltic fissure in relation to the older rhyolitic domes which the eruption intersects. Note that the fissure does not pass through the centre of Wahanga dome. The position of the dyke here studied is indicated by a star (see key for details). *Hashed area* shows where breccia of the Wahanga dome is exposed in the studied field area. *Inset:* the position of Fig. 1 in the wider Taupo Volcanic Zone in the North Island of New Zealand. (A detailed map of all dyke exposures is in Nairn and Cole (1981))

The Tarawera Volcanic Complex is part of the southern Okataina Volcanic Centre in the Taupo Volcanic Zone and it consists of 11 rhyolite domes and associated pyroclastic deposits (Cole 1970a). The youngest four domes—Crater, Ruawahia, Tarawera and Wahanga dome (from oldest to youngest; Fig. 1)—were emplaced in the ∼AD 1315 Kaharoa eruption episodes (Nairn et al. 2004). The most recent eruption from the Tarawera Volcanic complex was a basaltic Plinian eruption that took place on 10 June AD 1886. It began in the northeast within the Wahanga dome and propagated ∼17 km along a fissure to the southwest, forming ≥100 m deep craters through the existing dome complex (Cole 1970a; Walker et al. 1984; Houghton et al. 2004; Sable et al. 2006, 2009; Carey et al. 2007). The eruption was fed by several en echelon dykes (Nairn and Cole 1981) and disrupted a large pre-existing geothermal system at the southwest end of the fissure (Houghton et al. 2004). The event lasted ∼5 h (Nairn and Cole 1981; Houghton et al. 2004) during which ∼0.8 km^3^ of chemically uniform, high-alumina basaltic magma was erupted (Houghton et al. 2004) at an estimated temperature ranging from 1,200 to 800 °C (temperature estimates based on experimental validation of preserved textures; Kennedy et al. 2010).

A conceptual model of the eruption, assembled from multi-scale analyses of proximal deposits, has outlined the following eruption stages (detailed in: Houghton et al. 2004; Sable et al. 2006; Carey et al. 2007): (1) An initial phase beginning with phreatomagmatic eruptions from magma-groundwater interactions and vent-opening explosions. The opening of vents led to more rapid ascent of vesiculating magma, a change in fragmentation depth and a shift to purely magmatic eruption. (2) Peak Plinian intensity was achieved at discrete discharge-focus sites along the vent system. (3) A waning stage of Plinian activity, followed by a discharge of actively vesiculating magma and accumulation of partially outgassed magma in the shallow plumbing system. These processes reduced the effective radius of the conduit and led to an increase in depth of fragmentation. (4) A further increased depth of fragmentation along with waning magma flux led to renewed interaction with groundwater, removing much of the partially-outgassed magma.

**Fig. 2:**
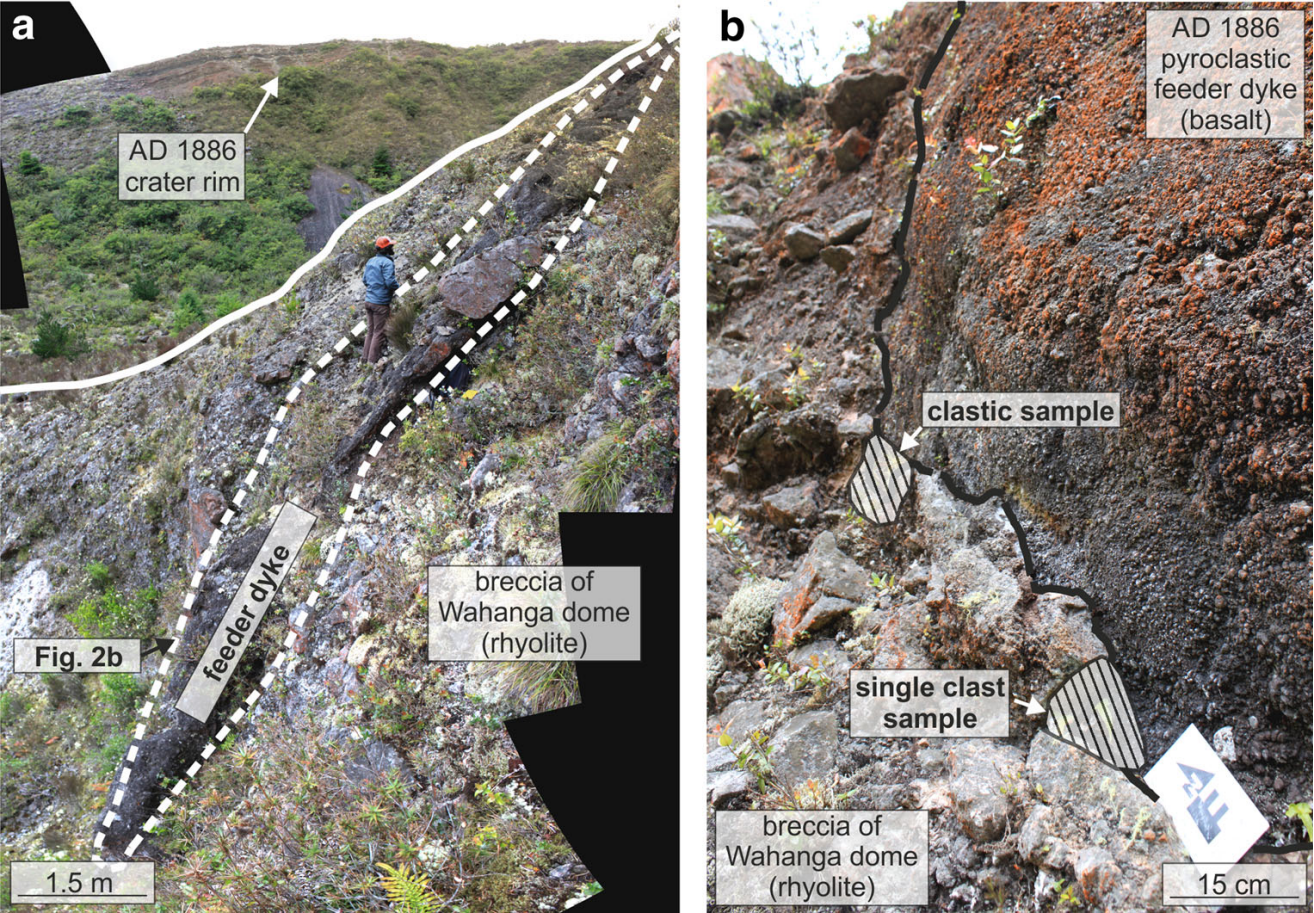
Outcrops relevant to this study. **a** A photograph showing the pyroclastic feeder dyke and the surrounding breccia of the Wahanga dome. **b** A photograph showing the marginal contact between the AD 1886 basaltic dyke and the Wahanga dome rhyolite on the SW side of the feeder dyke. Sample locations of the single clast sample and the clastic sample are highlighted

**Fig. 3:**
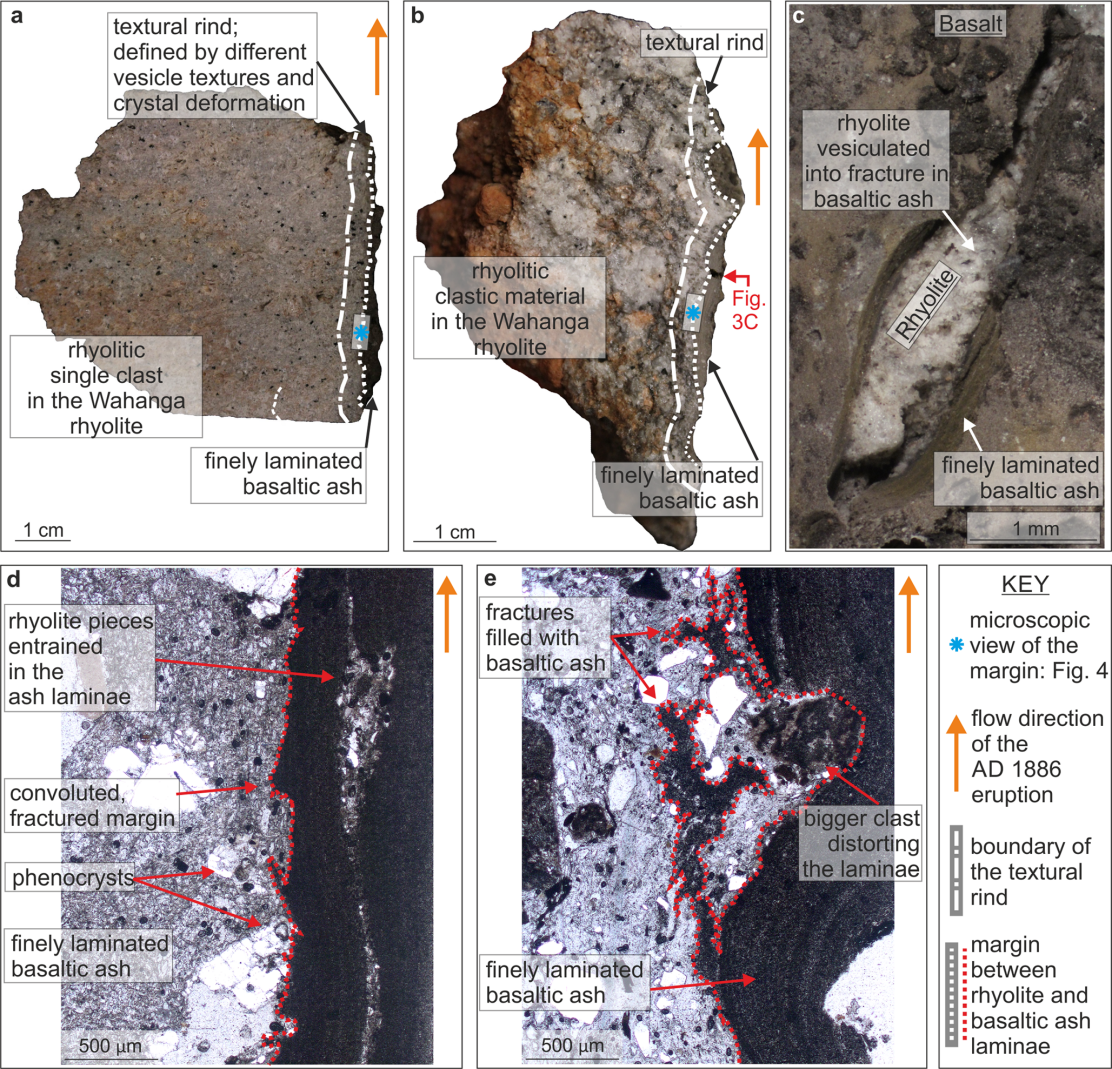
**a** Photograph of the single clast sample in contact with the finely laminated basaltic ash. The textural rind is highlighted. **b** Photograph of the indurated clastic rhyolite in contact with the feeder dyke margin featuring sintered basalt. The textural rind refers to the deformed domain at the marginal contact. **c** A close up of the sample in Fig. 3b of the dyke to rhyolite interface showing a crack in the sintered, finely laminated basalt and the vesiculated rhyolite exposed beneath. **d** Photomicrograph of the single clast sample and the adjacent basaltic ash. Domains of rhyolite are embedded in the basaltic ash. Note the highly convoluted and fractured margin. **e** Photomicrograph of the interface between clastic rhyolite and the laminated basaltic ash. The rhyolite contains fractures filled by basaltic ash. The contact between the two lithologies is convoluted and fractured and distorted by a bigger clast. The flow direction of the dyke material is indicated

The eruption is believed to have come to an end as the overpressure in the magma source dropped and magma-water interaction was no longer sufficiently extensive to sustain phreatomagmatic fragmentation. On the basis of mapping, Nairn and Cole (1981) have identified en echelon basaltic dykes along the AD 1886 Tarawera rift, which are now well-exposed in the base of the preserved eruption fissure. They were filled with basaltic scoria bombs, lapilli and ash after the eruption. Here, we describe one such dyke in the northeastern part of the fissure (dyke 2 from Nairn and Cole 1981). It varies between 4 and 6 m in width and is exposed at the surface of the Wahanga dome over a distance of 6 m from above and over 10 m height in cross section (Fig. 2). The dyke is entirely clastogenic. Close to the dyke margins the pyroclastic material within the dyke is dominated by ash sized particles that are finely laminated parallel to the margin on the scale of hundreds of micrometres to tens of millimetres (Fig. 3d–e). The rest of the dyke is thought to be backfilled with pyroclasts from the final phase of the AD 1886 eruption (Nairn and Cole 1981; Walker et al. 1984; Carey et al. 2007). The clast sizes grade into coarse bombs at >50 cm from the margin (Fig. 2a). The dyke is hosted by the AD 1315 Wahanga dome-forming rhyolite (Figs. 1 and 2), which is composed of clasts from 0.5 to 100 cm in diameter, cemented by indurated rhyolitic matrix fines (Fig. 2). The rhyolite is highly vesicular and brecciated near the dyke but intact in the walls of the dome that are exposed in the Tarawera fissure margins. At this site, there is no crystal alignment apparent in the rhyolite (Ashwell et al. 2015).

We undertook an in-field assessment of the lithologies exposed at the dyke to confirm the broad-scale features described in Kennedy et al. (2010) and Nairn and Cole (1981). Namely that there is a consistent deformed rind of marginal wall rock in contact with the outer surface of the dyke. This coherent rind is texturally homogeneous along strike of the dyke and implies heat transfer between basalt and rhyolite, at least on the scale of the outcrop (Kennedy et al. 2010). According to Nairn and Cole (1981) this rind is the dense baked margins of the rhyolite in contact with the dyke that is polished and striated. Similarly, we examined blocks of the rhyolite far from the marginal dyke contact (>10 m) to assess their variability and, while there are clast to clast differences, these samples are always texturally distinct from the deformed rind at the margin.

From the dyke margins, we collected a sample of (1) a locally representative ∼20 cm diameter rhyolitic single clast (Figs. 2b and 3a) and (2) a clastic rhyolitic sample, which consists of several smaller clasts agglutinated by finer-grained rhyolite (Figs. 2b and 3b–c). Both samples exhibit a distinct 0.1–3 cm thick and deformed rind at the rhyolite wall rock to basalt dyke interface representing syn-eruption melting of the host rhyolite and near-simultaneous sintering of some of the basaltic material (Fig. 3). For comparison, we also sampled 10–50 cm rhyolite dome breccia clasts with variable internal textures from the rhyolitic material at distance (>10 m from the conduit margin) to the AD 1886 feeder dyke. We limit our observations to the marginal contact between the basalt and the rhyolite, which represent syn-eruptive deposits passing by and sintering to the marginal rhyolite.

## Micro-scale textures

**Fig. 4:**
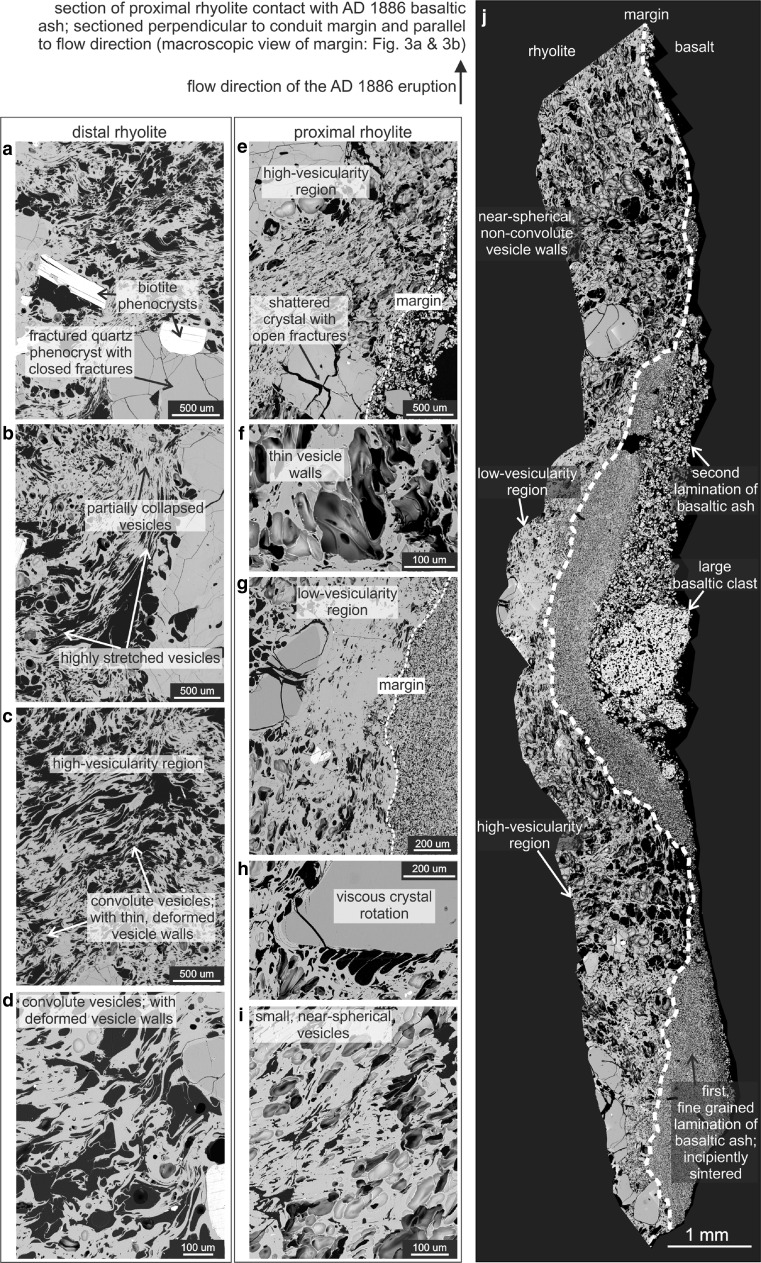
**a**–**d** Textures in the distal rhyolite shown in representative backscatter electron micrographs. **a** Fractured quartz phenocrysts with closed fractures. Biotite crystals show intact cleavage. **b** Vesicles range from partially collapsed to highly stretched and contorted. **c** High-vesicularity region with convolute vesicles which feature thin and deformed vesicle walls. **d** Convolute vesicles with deformed walls. **e**–**i** Textures in the proximal rhyolite (single clast and clastic material) shown in representative backscatter electron micrographs. **e** High-vesicularity region featuring a population of small, near-spherical vesicles. Crystals at the dyke interface generally have open fractures. **f** The vesicles in the textural rind have well-defined, thin walls. **g** Low-vesicularity region between the dyke interface and a fractured phenocryst. **h** Larger (∼0.1–0.2 mm) vesicles with preferential orientation of elongation in the wake of crystal rotation in the reheated rind. **i** Small and near-spherical vesicles in the rind stand in contrast to large and convolute vesicles in the distal rhyolite. **j** An overview section of the proximal rhyolite in contact with the AD 1886 basaltic ash; the rhyolite shows low- and high-vesicularity regions; two laminations of fine basaltic ash are present, disturbed by a big basaltic clast. All photos are taken perpendicular to the conduit margin and parallel to the flow direction of the AD 1886 eruption

**Fig. 5:**
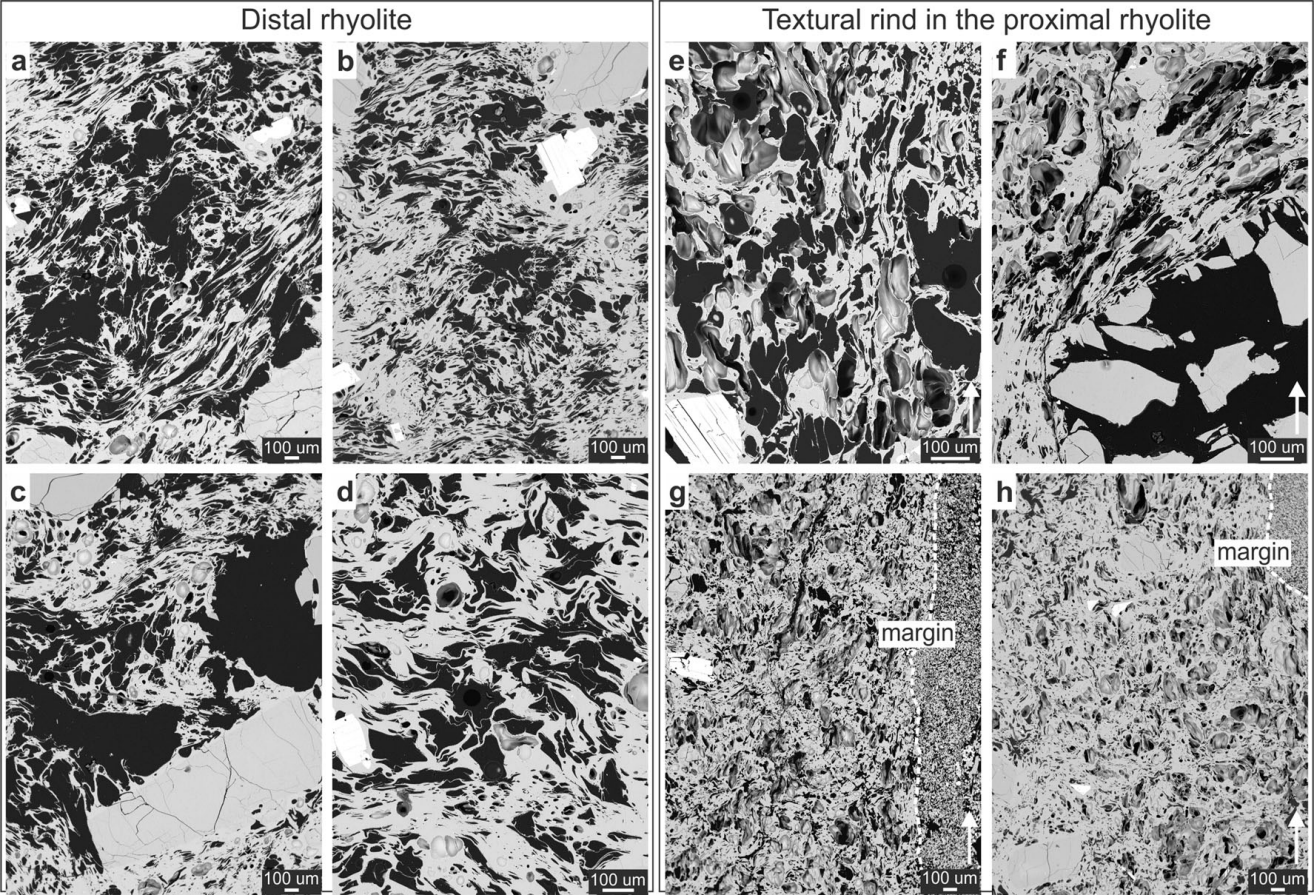
Comparison of the distal and proximal textures. **a**–**d** SEM images of the distal rhyolite. **a** Large, highly convoluted and stretched vesicles dominate the distal rhyolite. **b** A set of high- and low-vesicularity regions with intact phenocrysts. **c** Phenocrysts are fractured. **d** Vesicles are contorted. **e**–**h** SEM images of the proximal rhyolite. **e** Cleaved mica crystal in highly vesicular rhyolite. **f** Phenocryst in the rind are shattered and exhibit open fractures. **g**–**h** The textural rind is dominated by small-sized, near-spherical vesicles with well-defined vesicles walls population of smaller, spherical vesicles overprinting the distal textures. The dyke margin is indicated

Below we describe the textures of (1) a rhyolitic breccia clast situated at 10 m from the basaltic dyke margin, taken to represent the original rhyolitic textures, unmodified by the 1886 eruption (distal rhyolite; Figs. 4a–d and 5a–d) and (2) two oriented clasts of rhyolitic breccia at the margin of the AD 1886 basaltic pyroclastic dyke (proximal rhyolite; Figs. 3, 4e–j and 5e–h). Both rhyolitic breccias are from a flow lobe within the Wahanga dome. Thin sections of the oriented clasts were cut perpendicular to the strike of the dyke margin and parallel to the vertical flow direction, and our description of this strain localisation phenomenon focuses on vesicle textures and biotite deformation within a few millimetres of the dyke margin.

### Distal rhyolite wall rock

The porosity of the distal rhyolite is typically 28–30 vol.% (Kennedy et al. 2010) and is predominantly connected (Ashwell et al. 2015). Vesicle sizes vary over a large range from micron- to millimetre-sized and vesicle shapes are spherical to convolute and contorted, recording locally differential amounts of shear strain. There are domains of hundreds of microns to millimetres of relatively low (∼5 vol.%) vs. relatively high vesicularity (∼50 vol.%; Figs. 4a–d and 5a–d). In the low-vesicularity regions, the vesicles are preferentially orientated parallel to crystal margins, and vary from near circular to highly elongated with aspect ratios up to 50 in 2D cross-section (Fig. 4a–d). In the high-vesicularity regions the vesicles are more convolute, i.e. more tortuous (relative to those observed in the low-vesicularity regions), and the thin vesicle walls are contorted, such that they intrude neighbouring vesicles (Fig. 4a–d). With increasing vesicle size, the vesicle geometry gets more complex.

The mineralogy of the distal rhyolite is dominated by plagioclase, quartz and biotite phenocrysts with minor components of opaque minerals, orthopyroxene, hornblende, apatite and zircon (Richnow 1999). The crystal content is 23–25 vol.% in the vesicle-free rock (Cole 1970b; Richnow 1999), and glass content approximates 75 vol.%. The plagioclase phenocrysts are typically euhedral to subhedral and fractured (Fig. 5c). The quartz phenocrysts vary in size from 0.8 to 1.6 mm, and they feature closed fractures (i.e. the counterparts are adjacent to each other; Figs. 4a–b and 5a–b). The biotite phenocrysts are <0.5 mm, euhedral to subhedral and commonly fractured (Figs. 4a and 5b). Despite the fractures, the individual pieces of the crystal remain contiguous and show no apparent bending. Furthermore, the phenocrysts do not exhibit preferential orientation on the observed scale. See Electronic supplementary material (ESM) Fig. S2 for measurements of the groundmass glass of the distal rhyolite.

### Proximal rhyolite wall rock

The contact of the rhyolitic wall rock and the basaltic dyke (Figs. 3, 4e–j and 5e–h) is tortuous and disrupted, with fractures that are locally filled with basaltic ash, both parallel and perpendicular to the margin in the rhyolite wall rock (Fig. 3d–e). Pieces of rhyolite are entrained in the basaltic ash laminae (Fig. 3d). There is a ∼2-mm wide rhyolitic rind at the interface between the dyke and the host rock, which can be distinguished from the rhyolite wall rock by distinct vesicle textures and increased incidence and magnitude of crystal deformation (Figs. 3c–e and 4e–j, 5e–h). The rind features background vesicle textures broadly similar to those observed in the distal rhyolite, which are overprinted by a population of near-spherical, simple vesicles with well-defined vesicle walls (Figs. 4e–j and 5e–h). Such vesicle textures are not present in the unmodified part of the proximal rhyolite, nor in the distal rhyolite (Figs. 4a–d and 5a–d). These regions of spherical vesicularity are coincident with inflated fractures preserved in the adhered basaltic granular laminae (Fig. 3c).

The rind of the proximal rhyolite also hosts regions of low- and high-vesicularity, which are significantly larger in extent and with greater variation in vesicle size than any regions observed in the distal rhyolite. Some low-vesicularity regions are located where larger clasts of basalt are present very close to the margin and where the contact protrudes into the rhyolite (Fig. 4j). However, this is a rare feature. The vesicles are less deformed in regions of low-vesicularity relative to those of high-vesicularity regions, ranging from spherical to elongated with aspect ratios <10 in the latter. In at least one location at the edge of a 0.5- to 1-mm quartz phenocryst, large ∼0.1-to 0.2-mm vesicles exhibit preferential orientation of elongation at a common angle to the crystal edge (Fig. 4h), implying rotation of the crystal in a viscous melt. See ESM Fig. S2 for measurements of oxide distributions in the textural rind.

**Fig. 6:**
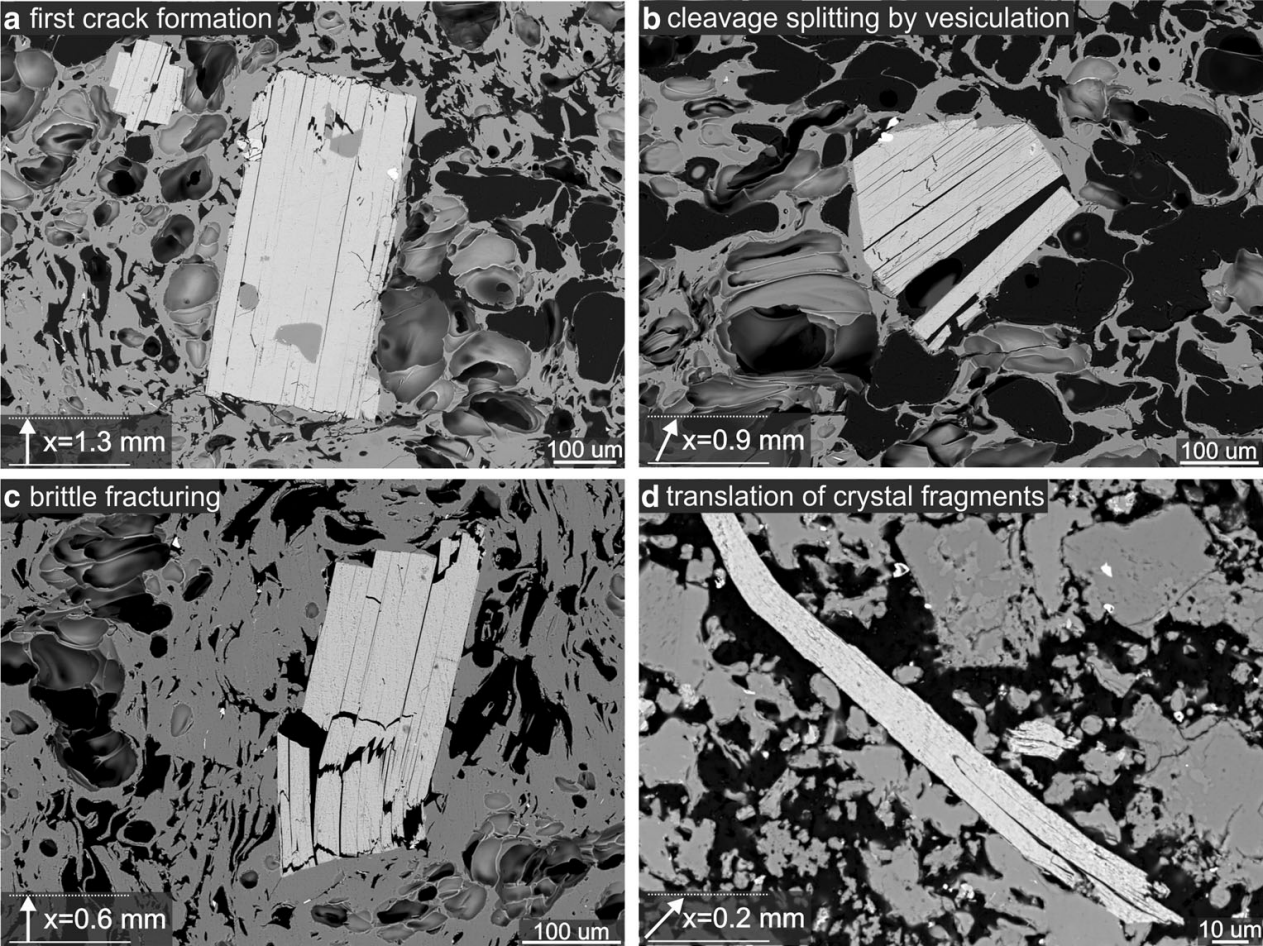
**a**–**d** The different deformation textures of biotite phenocrysts in the proximal rhyolite at decreasing distance (*x*) from the margin. Distance and direction to the margin are indicated by *arrows*; flow direction is perpendicular to the distance *arrow* and from *left to right*. **a** At a distance of 1.3 mm, first cracks are observed in the biotites. Pieces of the crystal remain contiguous. **b** At a distance of 0.9 mm, the cleavage is split by vesiculation of the groundmass glass. **c** At a distance of 0.6 mm, the phenocryst is pervasively fractured perpendicular to the cleavage and the mechanism is purely brittle. Bookshelf-like displacement is apparent. **d** At a distance of 0.2-mm single crystal fragments are translated and the cleavage is bent

**Fig. 7:**
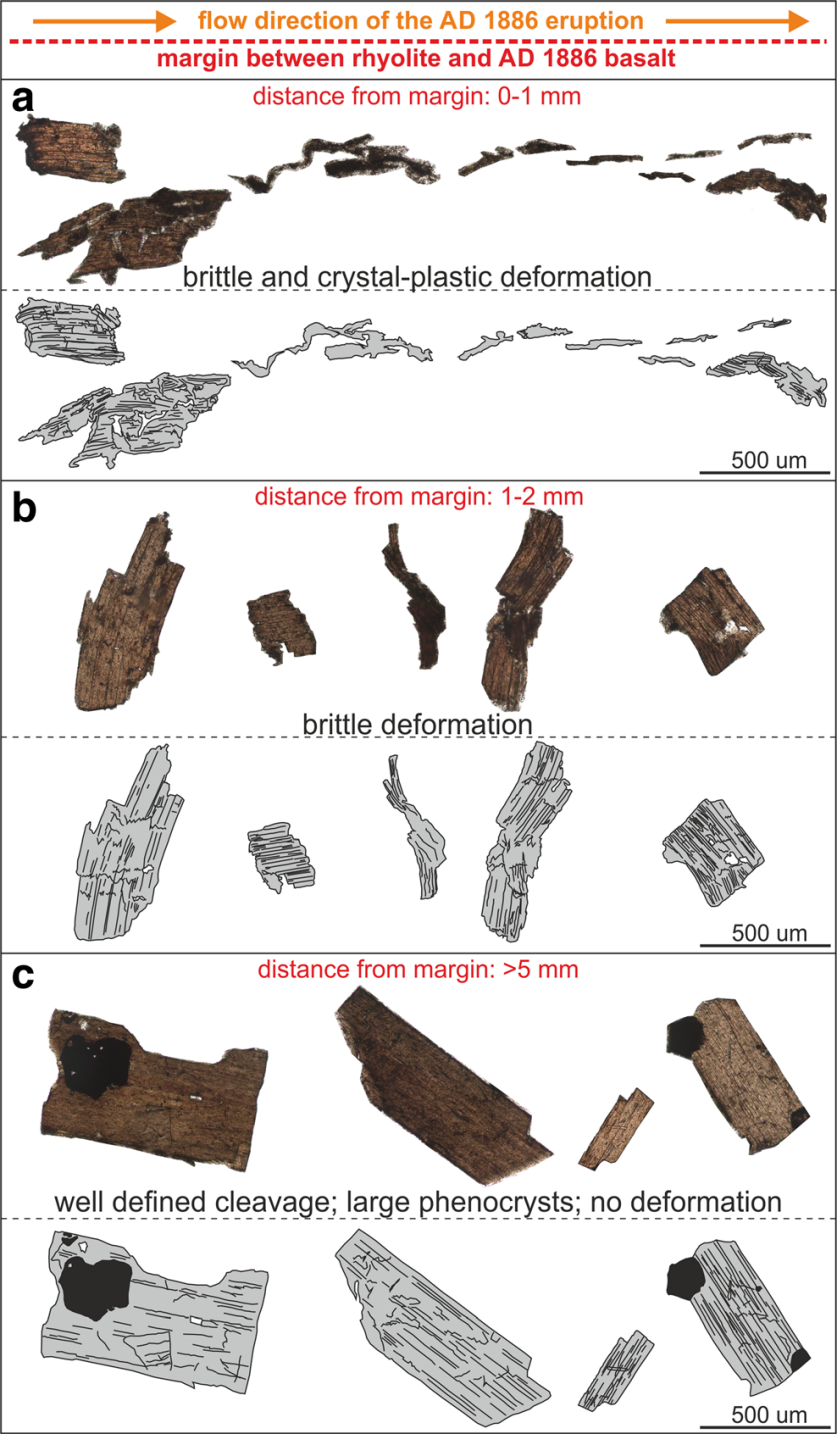
The variability of representative biotite textures in the textural rind in contact with the basalt feeder dyke (1–2 mm) compared with those from the distal rhyolite (>5 mm). Discrete crystals in this catalogue are to scale, but the inter-crystal distances are not. **a** At a distance of 0–1 mm from the margin, the deformation mechanism is crystal-plastic as well as brittle. The phenocrysts are deformed in a bookshelf-like fashion and single crystal fragments are separated and translated parallel to the margin, resulting in en echelon features. **b** At a distance of 1–2 mm from the margin, bookshelf-like displacements are apparent. Biotites with their cleavage oriented perpendicular to the margin, are deformed by cross-cleavage fracturing. The deformation mechanism is purely brittle. **c** At a distance of >5 mm from the margin, the crystals exhibit pristine cleavage and do not show similar deformation features compared to those crystals in the textural rind. The crystals lack cross-cleavage fractures and displacement of single fragments

The mineralogy of the rind of the proximal rhyolite is indistinguishable from that of the distal rhyolite. However, the shattered quartz and plagioclase phenocrysts in the rind feature open fractures, in contrast to closed fractures in the distal rhyolite (Figs. 4e, g and 5f). In addition, the biotite phenocrysts, described below, are deformed internally (Figs. 6 and 7a–b).

### The biotite phenocrysts in the distal and proximal rhyolite

In the distal rhyolite, the biotite phenocrysts are euhedral, tabular and exhibit pristine basal cleavage (Fig. 7c) as demonstrated by Richnow (1999) and Ashwell (2013). Broken crystals, which can be attributed to flow-induced stresses during dome growth (Allen and McPhie 2003) do not feature open fractures, the individual pieces are contiguous (Figs. 4a–b and 5a–c).

The biotites in the proximal rhyolite are different. Within the first few millimetres of the contact with the basalt, the biotite phenocrysts are deformed and biotite fragments are displaced (Figs. 6 and 7a–b). In the ∼2-mm thick rhyolite rind, biotite crystals dominantly exhibit brittle fractures and bookcase-style deformation along the basal cleavage planes (Figs. 6 and 7a–b). In the outermost 1 mm, the biotites are highly fractured and cleaved, and the cleavage planes vary, exhibiting undulose extinction (Figs. 6b–d and 7a). Where some minor bending of the crystal hast taken place, intracrystalline misorientation of the cleavage (in the 2D thin section plane) never exceeds 10°–15°. However, edge-to-edge fracturing and dislodging of biotite phenocrysts can result in subsequent rotation of one or both of the biotite pieces, creating an apparent angle that can exceed 15°.

The biotite phenocrysts in the single clast sample from the proximal rhyolite have been cleaved and in some instances the crystals are split along the cleavage planes with a measurable separation of the segments on either side of the plane. Each high-aspect-ratio-crystal fragment has been translated parallel to the margin resulting in en echelon features (Fig. 7a). This texture is absent in the clastic sample of the proximal rhyolite. Phenocrysts with their cleavage parallel to the margin are generally deformed by bookshelf-like displacements or separated along their cleavage planes (Fig. 7) whereas phenocrysts with their cleavage normal to the margin are fractured and show a large variation in basal cleavage orientation within a single crystal (Fig. 7).

**Fig. 8:**
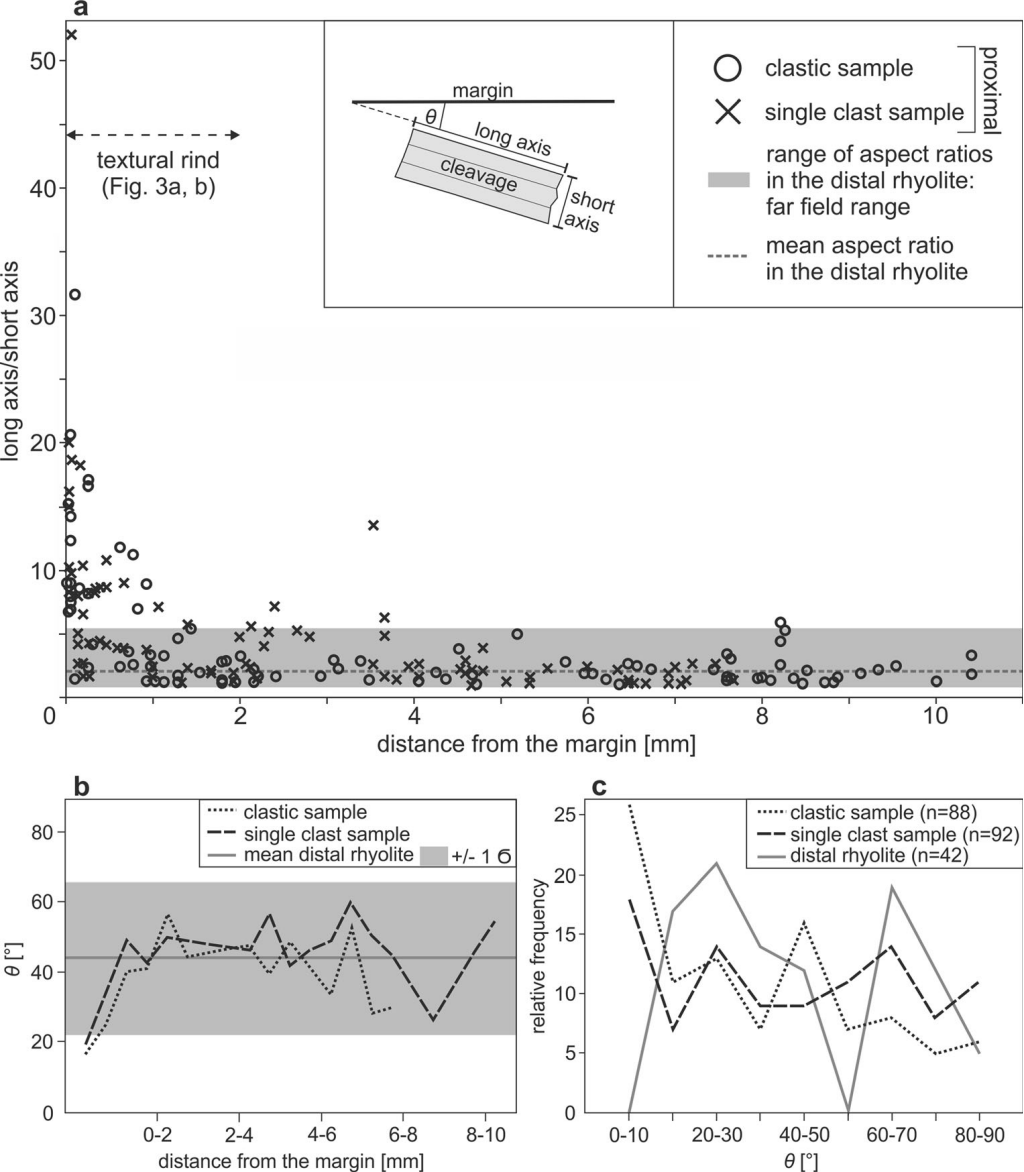
**a** The variability of aspect ratios of biotite in the clastic material and the single clast sample as a function of distance from the dyke to rhyolite interface compared to the aspect ratios of biotite in the distal rhyolite. Note the increase in the aspect ratios at a distance from the margin of ∼2 mm. The sketch shows how the aspect ratio data was obtained. The extent of the textural rind, is indicated by the *arrow*. **b** Distribution of *Θ* values in the clastic and the single clast sample as a function of distance to the margin shown in 0.5-mm bins. Mean value of the distal rhyolite and standard deviation are indicated for comparison. **c** Relative frequency of *Θ* values in the two whole proximal samples (clastic and single clast sample) and the distal rhyolite (distal rhyolite values are measured with respect to an arbitrary plane) shown in 10° bins

The distal rhyolite biotites have aspect ratios of 1.0–5.6 with a mean of 1.9 and a standard deviation from the mean of 1.0 (grey shaded region Fig. 8a; Table S1). The proximal rhyolite shows similar biotite aspect ratios at >2 mm from the margin with a mean aspect ratio of 2.5 and a standard deviation of 1.8 (Fig. 8a; Table S1). However, at <2 mm of the margin, the aspect ratio of biotites in the proximal rhyolite increases in a non-linear fashion to maxima of 31.6 (single clast sample) and 52.0 (clastic sample) respectively, with mean values of 6.7 and 8.5 and standard deviations of 6.4 and 8.8 (Fig. 8a; Table S1). The maximum visible offset from the first to the last piece of a biotite phenocryst is 1.12 mm. The longest piece of that margin-parallel biotite phenocryst is 0.36 mm, which we take to be the approximate undeformed length, *L*_0_. Strain is (*L* − *L*_0_)/*L*_0_, where *L* is the undeformed length plus the maximum offset distance, which is 3.1 for this deformed biotite.

In the proximal rhyolite, at distances <1 mm from the margin with the basaltic feeder dyke, there is an increase in biotite phenocrysts with low *θ* values (*θ* is defined as the angle between the long axis of a biotite phenocryst and the conduit margin; Figs. 8b–c; Table S1). *θ* values in the distal rhyolite were measured to an arbitrary but fixed reference line, representing the main direction of the margin (the measurement orientation was adjusted to the margin orientation). Only crystals with their *c* axis parallel to the plane of section are included in this assessment. The distal rhyolite biotites have a mean *θ* of 44° with a standard deviation of 22° (grey-shaded region; Fig. 8b; Table S1). The proximal rhyolite biotites in both samples at distance >1 mm from the rhyolite-dyke interface have a mean *ϴ* of 49° with a standard deviation of 8° (Table S1). However, at distances <1 mm from the interface, the mean value of *θ* decreases to 24° with a standard deviation of 13° (Table S1). The relative frequency of angles <10° is significantly higher in both samples from the proximal compared to that of the distal rhyolite (Fig. 8c). Despite this evidence for physical modification of the biotites in the rind closest to the rhyolite–basalt interface, there is no sign of chemical modification in these biotite crystals (the compositional variability of biotites in the proximal and distal rhyolite in a ternary AFM diagram determined by EMPA is shown in ESM Fig. S1).

## The feasibility of reheating a conduit margin: a 1D solution to the heat equation

Diffusion of heat in a medium can be cast in one dimension and in Cartesian coordinates using

1 $$ \frac{\partial T}{\partial t}=\frac{\partial }{\partial x}\left(D\frac{\partial T}{\partial x}\right), $$

where *T* is the temperature, *t* is the time after the onset of diffusion, *x* the distance from the margin, and *D* the thermal diffusivity in the medium. Here, we consider pure heat conduction through a homogeneous rhyolitic country rock. The thermal diffusivity in rhyolitic material (glass or liquid) is a function of temperature (Bagdassarov and Dingwell 1994) and can be approximated by

2 $$ D={D}_0 \exp \left(\alpha T\right), $$

if *D*_0_ is an extrapolated diffusivity at *T* = 0 and *α* is a constant. We use *D*_0_ = 3.66 ⋅ 10^− 7^ m^2^·s^−1^ and *α* = 1.24 ⋅ 10^− 3^K^−1^ where the values of *T* are given in degree Celsius and not kelvin (see ESM for the relevant calibration). For a vesicular rhyolite, we scale the diffusivity calculated by Eq. 2 with the bulk porosity, following Connor et al. (1997), such that:

3 $$ D=\frac{k}{\rho C\left(1-\phi \right)+\rho^{\prime }C^{\prime}\phi }, $$

where *k* is the bulk thermal conductivity, *ρ* and *ρ* ′ are the density of the solid and pore fluid, respectively. *C* and *C* ′ are the specific heat capacity of the solid and pore fluid, respectively, and *ϕ* is the bulk porosity (expressed as a volume fraction). The thermal conductivity can also be scaled with porosity using the empirical model by Bagdassarov and Dingwell (1994) which relates *k* to its pore-free value *k*_0_ via

4 $$ k={k}_0\left(\frac{1-\phi }{1+\phi}\right), $$

For *ϕ* = 0.5, *D*_0_ = 1.626 ⋅ 10^− 7^ m^2^·s^−1^ and *α* is unchanged. We use typical density and heat capacity values for rhyolite glass or liquid of *ρ* = 2, 350 kg·m^−3^ and *C* = 1, 000 J·kg^−1^·K^−1^. The density and heat capacity of the atmosphere is taken to be *ρ* ′ = 1.275 kg·m^−3^ and *C* ′ = 1, 007 J·kg^−1^·K^−1^ (Lavallée et al. 2015). If we assume that initially the country rock is in thermal equilibrium at *T*_*i*_ (here taken to be ∼20 °C), the initial condition can be described as follows:

5 $$ T={T}_i\ \mathrm{f}\mathrm{o}\mathrm{r}\ \mathrm{all}\ \mathrm{values}\ \mathrm{o}\mathrm{f}\ x\ \mathrm{at}\ t=0\ . $$

At the rhyolite–basalt interface, we assume a constant wall temperature *T*_*w*_ (constant Dirichlet boundary condition) of

6 $$ T={T}_w\ \mathrm{f}\mathrm{o}\mathrm{r}\ t>0\  at\ x=0, $$

And in the far field (i.e. *x* → ∞), we assume that the boundary is insulated (Neumann boundary condition of 0)

7 $$ \frac{\partial T}{\partial x}=0\ \mathrm{f}\mathrm{o}\mathrm{r}\ t>0\  at\ x=L, $$

where *L* is some depth into the country rock which represents the far field.

By solving Eq. 1 numerically using a finite difference explicit forward-time, central-space scheme, we have the tools necessary to model the evolution of temperature in rhyolite country rock with any changing temperature at the margin.

**Fig. 9:**
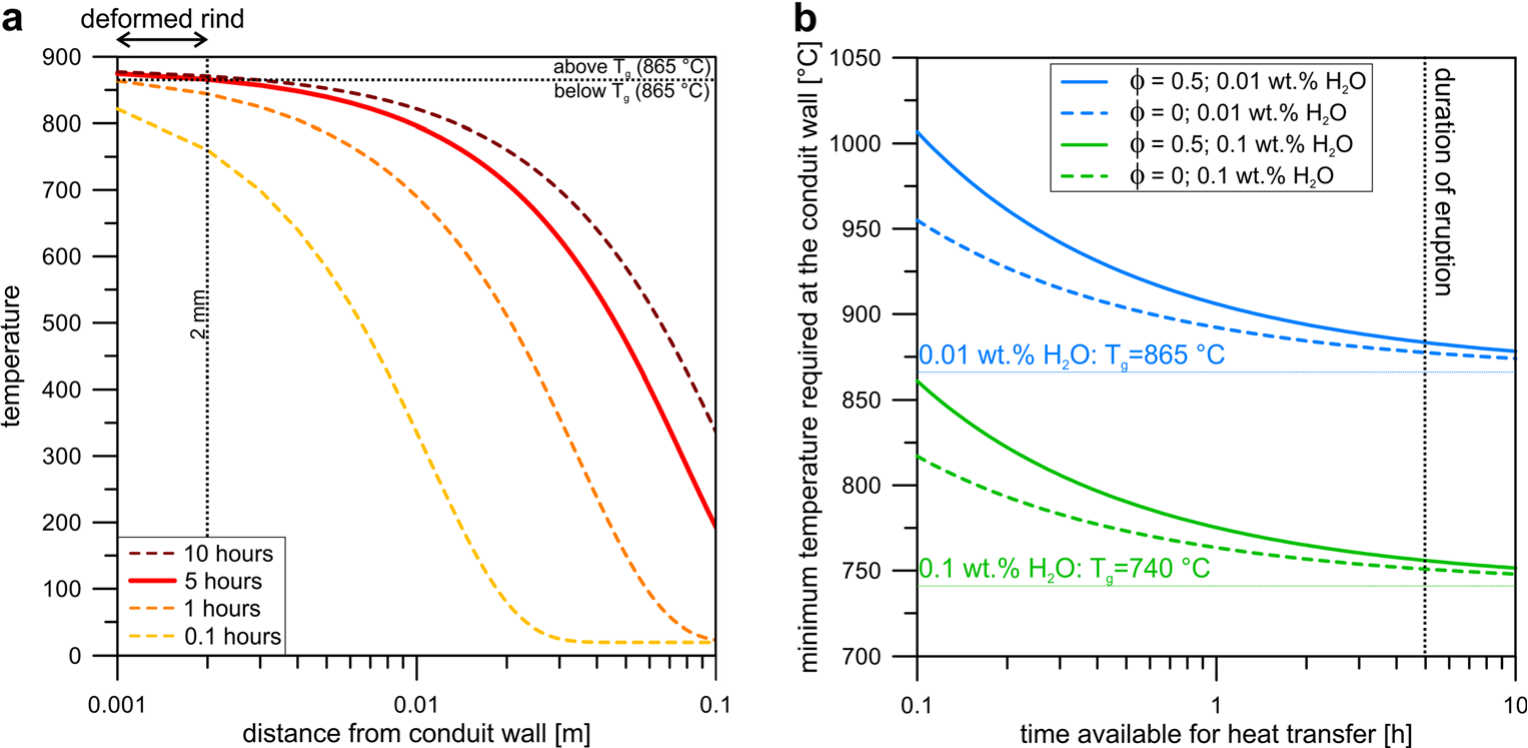
**a** Temperature profiles in the wall rock at a constant boundary temperature condition for 10, 5, 1 and 0.1 h, at a porosity of *ϕ* = 0.5 and a dissolved water content of 0.01 wt.% . The glass transition temperature *T*_g_ of ∼865 °C is approximated by a melt viscosity of 10^11.4^ Pa·s using Hess and Dingwell (1996). The thickness of the textural rind of ∼2 mm is highlighted. The timescale of the AD 1886 eruption of ∼5 h is sufficient to heat the thickness of the textural rind above its glass transition when the boundary temperature is 880 °C. **b** Time available for heat transfer into the wall rock as a function of the minimum required temperature at the conduit wall to heat the textural rind (∼2 mm) to the glass transition temperature. Solutions are for dissolved water contents of 0.1 wt% (*T*_g_ = ∼740 °C) and 0.01 wt% (*T*_g_ = ∼865 °C) and porosities of *ϕ* = 0.5 and *ϕ* = 0. Eruption timescale of ∼5 h is highlighted. In all scenarios, the temperature at the dyke interface is lower than typical basaltic eruptive temperatures

We now use this model to assess the feasibility of heating the conduit wall by the advective passing of hot material inside the conduit during an eruption of similar duration as the 1886 Tarawera event. Explicit assumptions here are that: (1) the conduit margin is stationary such that no spalling or erosion of the margin occurs on the timescale of model/eruption and (2) any deformation of the margin is small such that translation of high or low porosity regions is not considered. We provide in Fig. 9a the solution to Eq. 1 for which the boundary temperature was the constant value required to reach a glass transition temperature of ∼865 °C (determined by the liquid viscosity at the glass transition of 10^11.4^ Pa·s for a dissolved total water content of 0.01 wt.% using the viscosity model of Hess and Dingwell 1996) at the spatial position defined by our observations (∼2 mm) in the time of the eruption (∼5 h; Nairn and Cole 1981; Houghton et al. 2004) when the porosity of the conduit wall is *ϕ* = 0.5. The glass transition is the minimum temperature below which any vesiculation or viscous deformation is impossible. Since these features are present in the textural rind, the boundary temperature of 865 °C found for this solution is also a minimum temperature. If we acknowledge that the time available for heat transfer could be less than the eruption duration of 5 h, we find that the conduit boundary temperature required to heat our texturally defined marginal zone to its glass transition is slightly higher (Fig. 9b). We use the viscosity model of Hess and Dingwell (1996), which accounts for the effects of dissolved H_2_O, to provide solutions for water contents of 0.01 and 0.1 wt.% (see Kennedy et al. 2010), which in turn shifts the position of the viscosity threshold above which we approximate the rhyolite as a glass. We also show results for the two end member porosities of 0 and 0.5 (estimated porosity range based on textural observations). The approximated glass transition viscosity (10^11.4^ Pa·s) occurs at temperatures 865 and 740 °C for 0.01 and 0.1 wt.% dissolved water, respectively. In all solutions provided, the obtained temperature is much lower than a typical eruptive basalt temperature (Fig. 9b), which is reasonable as the dyke is exposed above the fragmentation depth (fully pyroclastic) and so may well be preserving the cooler portion of the uppermost conduit. This result is consistent with estimates of margin temperature minima of 800 °C from Kennedy et al. (2010).

We conclude that this simple heat transfer consideration coupled with the textural observations provide tantalizing evidence that reheating and remelting of the conduit margin and that this could well have involved deformation above the glass transition and that indeed this process is physically feasible.

## Interpretation and discussion

### Wall rock textures: the distal to proximal facies

To interpret the textural evolution of the deformed rhyolite rind, it is critical to distinguish between textures in the rhyolite which are likely to be related to dome-forming processes during the ∼AD 1315 Kaharoa eruption and those formed during reheating and subsequent deformation at the conduit wall associated with the AD 1886 Plinian basaltic eruption. Dome-forming processes can impart a wide range of textures and such extrusion and emplacement textures have been reproduced experimentally, including bubble coalescence textures (e.g. dimple vesicle wall textures; Castro et al. 2012b), bubble collapse textures (Westrich and Eichelberger 1994; Kennedy et al. 2010) and bubble deformation textures (e.g. Rust and Manga 2002). In the Wahanga dome itself, vesicle orientation is highly variable and vesicles are always contorted and elongate (Ashwell 2013; Ashwell et al. 2015), as is typical for moderately porous volcanic domes (Manley and Fink 1987). Local flow complexities and local heterogeneities in groundmass vesicularity are attributable to strain localisation (Okumura et al. 2008; Wright and Weinberg 2009; Laumonier et al. 2011). Here, crystals in the distal sample have no preferred orientation (see “Distal rhyolite wall rock” section), and there is no preserved evidence for post dome emplacement deformation as described in section “Proximal rhyolite wall rock”.

In the vicinity of the basaltic conduit, the textures stand in stark contrast to that of the distal rhyolite, including evidence for phenocryst fractures, biotite deformation, secondary vesiculation and bubble collapse (see “Proximal rhyolite wall rock” section). We ascribe the textural difference to the high temperatures and stresses imposed by the erupting column on the rhyolitic conduit walls, which work in concert to generate strain in the conduit wall. The temperature of a basaltic eruption mixture exceeds the glass transition temperature of rhyolite and the juxtaposition of a large flux of hot basaltic granular magma and volcanic gases with the glassy rhyolitic dyke wall upon eruption therefore has the capacity to thermally remobilize the rhyolite (see “The feasibility of reheating a conduit margin: a 1D solution to the heat equation” section). As the dyke was packed with hot basaltic pyroclasts after the eruption, temperatures above the rhyolite glass transition were likely maintained longer than the eruption timescale of ∼5 h. The high proportion of groundmass glass in the rhyolite makes this initially solid phase thermally susceptible to “softening” and viscous deformation as the glass transition temperature is exceeded (Dingwell and Webb 1990)*.* In the case of a sustained eruptive flow, the lateral heat transfer will result in a time-dependent temperature gradient in the marginal wall rock (Carrigan et al. 1992). Hence, the “penetration” distance from the margin within which the groundmass glass can be viscously remobilised increases with time (Kennedy et al. 2010). Furthermore, within a sufficient eruptive duration, the temperature of the rhyolitic conduit wall near the contact is expected to increase rapidly to temperatures approaching the basaltic eruptive temperature (Carrigan et al. 1992). The modified rhyolite rind records evidence of both brittle and viscous deformation. This behaviour is highlighted by brittle (cleaved and fractured) and minor crystal-plastic deformation in biotite phenocrysts, whereby the crystals exhibit both undulose extinction and bent cleavage. Crystal rotation resulting in stretched vesicles cannot occur without the groundmass glass first relaxing to a viscous liquid.

### Biotite deformation in the wall rock

Within the deformed rhyolite rind, biotite crystals with their (001) cleavage approximately perpendicular to the margin show bending and fracturing textures, whereas biotite crystals with their (001) cleavage approximately parallel to the margin show cleavage steps and cleavage separation. Additionally, an accumulation of margin-parallel biotite crystals implies a rotation via flow. The maturity of these modifications increases with proximity to the margin, which is evidence for increasing shear strain (Kanaori et al. 1991). The calculated strain of 3.1 is a minimum because it is unlikely that a thin section suite captures a complete set of biotite fragments translated parallel to the margin. However, preparation of samples perpendicular to the strike of the dyke and parallel to the vertical flow direction optimises the possibility of accurately assessing strain. The absence of further deformation mechanisms in the rhyolite rind such as kink bands in the biotite crystals or recrystallized biotite grains—both of which are observed in cataclasites and mylonites (Bell 1979; Kanaori et al. 1991)—is here attributed to the short time-scale of the eruption. The short eruption time can also explain the absence of reaction rims in the textural rind and uniform chemical composition of the biotite crystals (ESM Fig. S1) in the proximal and the distal rhyolite.

### Vesiculation and diffusion

Increasing vesiculation towards the margin, recording a heating above the glass transition temperature of the rhyolite groundmass glass, where oversaturated volatiles started to diffuse and vesicles form, is a natural consequence of higher temperatures near the margin. The vesiculation event at the margin observed in this study is confined to the deformed rhyolite rind at the dyke interface. The vesicles are round and only partially coalesced, suggesting little or no shear deformation was accommodated in this region after the vesiculation event. Vesiculation also caused brittle inflationary cracks in the accumulated ash layers. This implies that vesiculation in the rhyolite continued after accumulation of sintered basaltic ash along the dyke margin, because for the crack to form, the laminated and partly welded ash had to cool below the basalt solidus. Thus, we propose that, while it is possible that brittle and ductile deformation as well as vesiculation processes were coeval and in close spatial proximity to each other, vesiculation continued for a period of time after the cessation of the eruption.

### Implications of conduit wall sealing

The thermal model reveals that the eruptive time of ∼5 h suffices to heat 2 mm of the rhyolitic wall rock, the thickness of the deformed rind, above the glass transition temperature. Given that the eruption duration represents a minimum time available for heat transfer and that the samples are from a shallow depth of ∼10 m, we suggest that our proximal rhyolite samples only represent a minimum of conduit deformation which could be more pervasive at depth. We expect the thickness of the deformation zone and the temperatures reached to be an increasing function of depth in the conduit. Therefore, at greater depth the process of conduit wall sealing by viscous compaction, or of conduit wall vesiculation by pervasive reheating may be even more significant. Conduit margin deformation has implications for conduit permeability and outgassing (Rust et al. 2004; Kennedy et al. 2010), potential conduit wall sealing (e.g. Kennedy et al. 2010) and the explosivity of large eruptions (e.g. Jaupart and Allègre 1991). We highlight that conduit-wall sealing may not be the dominant mechanism for the explosivity of the AD 1886 Tarawera eruption and that phreatomagmatism is clearly a critical additional factor (e.g. Houghton et al. 2004). However, we use our observations from this locality to infer the importance of this process at depth in volcanic interiors for which the conduit wall rocks contain large fractions of glass, which is easily remobilized on re-heating.

## Concluding remarks

Textural examination of a shallow feeder dyke reveals that the energetic AD 1886 basaltic Plinian eruption of Tarawera volcano caused reheating of the marginal wall rock such that (1) the groundmass glass softened to a viscously deformable liquid to form a 2-mm rhyolite rind at the dyke interface (“The feasibility of reheating a conduit margin: a 1D solution to the heat equation” section), (2) the groundmass is variably densified and vesiculated (“Micro-scale textures” section), (3) the biotite crystals in the rind are broken, rotated and deformed by viscous flow (“Micro-scale textures” section) and (4) brittle fractures occurred in the rhyolite wall rock (“Micro-scale textures” section). The data presented here highlight the pervasiveness of magma–rock interaction during explosive volcanic activity and its potential implications for magma degassing. Not measured here is the conduit wall permeability, for which significant changes in conduit wall porosity have implications. We conclude that a fruitful line of future inquiry would be to tie these textural constraints and the experimental constraints of Kennedy et al. (2010) with a suite of systematic measurements of permeability to assess whether conduit wall sealing may be a mechanism for preserving high overpressure required for large basaltic Plinian activity.

## Electronic supplementary material

Below is the link to the electronic supplementary material.

ESM 1 (DOCX 387 kb)
