# Supplementary material for: Conduit margin heating and deformation during the AD 1886 basaltic Plinian eruption at Tarawera volcano, New Zealand
Source: Bull Volcanol. 2016 Feb 13;78:12. doi: 10.1007/s00445-016-1006-7 (PMC4913976; doi:10.1007/s00445-016-1006-7)

**Conduit margin heating and deformation during the AD 1886 basaltic Plinian eruption at Tarawera volcano, New Zealand**

**---Supplementary information---**

Jenny Schauroth^1,*^, Fabian B. Wadsworth^1^, Ben Kennedy^2^, Felix W. von Aulock^3^, Yan Lavallée^3^, David E. Damby^1^, Jérémie Vasseur^1^, Bettina Scheu^1^, Donald B. Dingwell^1^

^1^ Department of Earth and Environmental Science, Ludwig-Maximilians-Universität (LMU), Theresienstr. 41, 80333 Munich, Germany.

^2^ Department of Geology, University of Canterbury, Private Bag 4800, Christchurch, New Zealand.

^3^ School of Earth, Ocean and Ecological Science, University of Liverpool, Browlow Street, Liverpool, United Kingdom

* Corresponding author: [jenny.schauroth@min.uni-muenchen.de](mailto:jenny.schauroth@min.uni-muenchen.de)

1. **The raw biotite geometry and orientation data**

Table S1 provides the raw data for the biotites measured here in pertaining to Figure 8 in the main article. The long and short axis of mica phenocrysts in the distal and proximal samples, the resulting aspect ratio and angle of orientation *ϴ* (measured with respect to the margin in the proximal sample and measured to a fixed, arbitrary plane in the distal sample).

Table S1

| **distal rhyolite** | | | | | **proximal breccia clast** | | | | | **proximal interclast material** | | | | |
| --- | --- | --- | --- | --- | --- | --- | --- | --- | --- | --- | --- | --- | --- | --- |
| **n** | **L [mm]** | **b [mm]** | **L/b** | **θ [°]** | **n** | **L [mm]** | **b [mm]** | **L/b** | **θ [°]** | **n** | **L [mm]** | **b [mm]** | **L/b** | **θ [°]** |
| 1 | 0.2 | 0.2 | 1.3 | 39 | 1 | 0.4 | 0.2 | 2.6 | 67 | 1 | 0.1 | 0.0 | 5.8 | 24 |
| 2 | 0.3 | 0.1 | 3.1 | 79 | 2 | 0.1 | 0.1 | 1.2 | 30 | 2 | 0.1 | 0.1 | 1.1 | 10 |
| 3 | 0.2 | 0.2 | 1.1 | 29 | 3 | 0.2 | 0.1 | 1.5 | 1 | 3 | 0.1 | 0.1 | 1.1 | 43 |
| 4 | 0.1 | 0.1 | 1.6 | 77 | 4 | 0.1 | 0.0 | 2.6 | 79 | 4 | 0.1 | 0.1 | 1.3 | 44 |
| 5 | 0.2 | 0.1 | 1.6 | 41 | 5 | 0.4 | 0.1 | 5.0 | 42 | 5 | 0.1 | 0.0 | 2.2 | 55 |
| 6 | 0.1 | 0.1 | 2.3 | 67 | 6 | 0.2 | 0.2 | 1.1 | 50 | 6 | 0.1 | 0.0 | 1.5 | 48 |
| 7 | 0.2 | 0.1 | 2.1 | 24 | 7 | 0.2 | 0.1 | 2.6 | 19 | 7 | 0.0 | 0.0 | 2.3 | 55 |
| 8 | 0.2 | 0.2 | 1.1 | 47 | 8 | 0.1 | 0.1 | 1.5 | 85 | 8 | 0.1 | 0.0 | 1.1 | 16 |
| 9 | 0.1 | 0.1 | 1.1 | 31 | 9 | 0.2 | 0.1 | 2.3 | 63 | 9 | 0.1 | 0.0 | 1.4 | 17 |
| 10 | 0.3 | 0.2 | 1.1 | 50 | 10 | 0.2 | 0.1 | 2.4 | 69 | 10 | 0.1 | 0.1 | 1.1 | 1 |
| 11 | 0.2 | 0.1 | 2.8 | 66 | 11 | 0.2 | 0.1 | 3.6 | 41 | 11 | 0.0 | 0.0 | 1.9 | 86 |
| 12 | 0.1 | 0.1 | 1.1 | 84 | 12 | 0.3 | 0.0 | 11.2 | 23 | 12 | 0.5 | 0.2 | 2.7 | 36 |
| 13 | 0.1 | 0.1 | 1.2 | 40 | 13 | 0.1 | 0.1 | 1.2 | 67 | 13 | 0.0 | 0.0 | 2.2 | 48 |
| 14 | 0.2 | 0.1 | 1.2 | 38 | 14 | 0.3 | 0.0 | 20.6 | 10 | 14 | 0.1 | 0.0 | 4.3 | 55 |
| 15 | 0.2 | 0.1 | 1.8 | 64 | 15 | 0.2 | 0.0 | 7.5 | 10 | 15 | 0.1 | 0.0 | 4.3 | 2 |
| 16 | 0.3 | 0.2 | 1.3 | 29 | 16 | 0.6 | 0.0 | 16.6 | 7 | 16 | 0.1 | 0.0 | 2.7 | 30 |
| 17 | 0.2 | 0.1 | 2.3 | 17 | 17 | 0.2 | 0.0 | 8.5 | 27 | 17 | 0.1 | 0.0 | 8.0 | 4 |
| 18 | 0.2 | 0.1 | 1.3 | 23 | 18 | 0.4 | 0.1 | 2.8 | 73 | 18 | 0.1 | 0.0 | 3.8 | 1 |
| 19 | 0.3 | 0.1 | 3.3 | 16 | 19 | 0.1 | 0.1 | 1.1 | 78 | 19 | 0.1 | 0.1 | 1.7 | 42 |
| 20 | 0.5 | 0.1 | 3.8 | 28 | 20 | 0.1 | 0.0 | 2.4 | 34 | 20 | 0.1 | 0.0 | 4.8 | 86 |
| 21 | 0.1 | 0.1 | 1.1 | 65 | 21 | 0.4 | 0.2 | 2.1 | 10 | 21 | 0.1 | 0.0 | 2.9 | 2 |
| 22 | 0.1 | 0.1 | 1.2 | 29 | 22 | 0.1 | 0.0 | 3.0 | 58 | 22 | 0.1 | 0.0 | 2.1 | 72 |
| 23 | 0.4 | 0.2 | 2.1 | 13 | 23 | 0.1 | 0.1 | 1.4 | 51 | 23 | 0.1 | 0.0 | 52.0 | 3 |
| 24 | 0.2 | 0.2 | 1.2 | 19 | 24 | 0.1 | 0.1 | 1.9 | 27 | 24 | 0.1 | 0.1 | 1.1 | 45 |
| 25 | 0.2 | 0.1 | 2.5 | 83 | 25 | 0.2 | 0.1 | 2.8 | 56 | 25 | 0.1 | 0.0 | 1.3 | 49 |
| 26 | 0.1 | 0.1 | 1.2 | 72 | 26 | 0.1 | 0.1 | 2.2 | 56 | 26 | 0.1 | 0.0 | 7.2 | 41 |
| 27 | 0.1 | 0.1 | 1.3 | 61 | 27 | 0.2 | 0.1 | 1.3 | 51 | 27 | 0.1 | 0.0 | 1.9 | 28 |
| 28 | 0.2 | 0.1 | 1.5 | 44 | 28 | 0.1 | 0.1 | 1.5 | 26 | 28 | 0.1 | 0.1 | 2.7 | 25 |
| 29 | 0.2 | 0.2 | 1.2 | 65 | 29 | 0.1 | 0.0 | 3.4 | 48 | 29 | 0.2 | 0.1 | 1.3 | 15 |
| 30 | 0.3 | 0.1 | 2.9 | 12 | 30 | 0.1 | 0.0 | 5.9 | 66 | 30 | 0.1 | 0.1 | 1.0 | 11 |
| 31 | 0.1 | 0.1 | 1.0 | 40 | 31 | 0.1 | 0.0 | 5.3 | 67 | 31 | 0.2 | 0.1 | 2.4 | 57 |
| 32 | 0.3 | 0.1 | 2.8 | 29 | 32 | 0.1 | 0.1 | 1.3 | 51 | 32 | 0.1 | 0.0 | 4.1 | 9 |
| 33 | 0.3 | 0.2 | 1.7 | 23 | 33 | 0.1 | 0.1 | 1.3 | 13 | 33 | 0.2 | 0.0 | 4.4 | 9 |
| 34 | 0.5 | 0.1 | 3.8 | 45 | 34 | 0.1 | 0.0 | 1.3 | 58 | 34 | 0.1 | 0.0 | 2.7 | 8 |
| 35 | 0.1 | 0.1 | 1.2 | 39 | 35 | 0.2 | 0.1 | 1.7 | 80 | 35 | 0.1 | 0.0 | 1.7 | 88 |
| 36 | 0.1 | 0.0 | 3.0 | 14 | 36 | 0.2 | 0.1 | 1.2 | 8 | 36 | 0.1 | 0.1 | 1.1 | 87 |
| 37 | 0.2 | 0.2 | 1.2 | 22 | 37 | 0.1 | 0.1 | 1.7 | 83 | 37 | 0.2 | 0.1 | 1.6 | 6 |
| 38 | 0.1 | 0.1 | 1.3 | 63 | 38 | 0.1 | 0.1 | 1.6 | 83 | 38 | 0.1 | 0.0 | 6.6 | 1 |
| 39 | 0.3 | 0.1 | 3.4 | 69 | 39 | 0.0 | 0.0 | 1.0 | 40 | 39 | 0.2 | 0.0 | 18.2 | 8 |
| 40 | 0.2 | 0.1 | 1.7 | 71 | 40 | 0.1 | 0.1 | 1.6 | 36 | 40 | 0.3 | 0.1 | 2.2 | 0 |
| 41 | 0.2 | 0.2 | 1.1 | 77 | 41 | 0.1 | 0.0 | 15.3 | 6 | 41 | 0.1 | 0.0 | 3.9 | 32 |
| 42 | 0.2 | 0.0 | 5.6 | 15 | 42 | 0.1 | 0.0 | 3.2 | 50 | 42 | 0.2 | 0.0 | 5.2 | 44 |
|  |  |  |  |  | 43 | 0.2 | 0.1 | 2.2 | 74 | 43 | 0.1 | 0.0 | 1.3 | 71 |
|  |  |  |  |  | 44 | 0.2 | 0.1 | 1.8 | 61 | 44 | 0.1 | 0.0 | 1.9 | 18 |
|  |  |  |  |  | 45 | 0.1 | 0.0 | 1.4 | 5 | 45 | 0.1 | 0.0 | 2.4 | 83 |
|  |  |  |  |  | 46 | 0.2 | 0.1 | 1.3 | 50 | 46 | 0.1 | 0.0 | 5.6 | 49 |
|  |  |  |  |  | 47 | 0.3 | 0.1 | 1.9 | 74 | 47 | 0.1 | 0.0 | 4.8 | 45 |
|  |  |  |  |  | 48 | 0.2 | 0.1 | 1.7 | 21 | 48 | 0.1 | 0.0 | 4.8 | 80 |
|  |  |  |  |  | 49 | 0.3 | 0.1 | 2.2 | 51 | 49 | 0.1 | 0.1 | 1.9 | 72 |
|  |  |  |  |  | 50 | 0.0 | 0.0 | 1.4 | 86 | 50 | 0.2 | 0.1 | 1.7 | 5 |
|  |  |  |  |  | 51 | 0.1 | 0.0 | 8.2 | 15 | 51 | 0.1 | 0.0 | 5.1 | 1 |
|  |  |  |  |  | 52 | 0.3 | 0.2 | 1.5 | 63 | 52 | 0.1 | 0.0 | 5.3 | 44 |
|  |  |  |  |  | 53 | 0.2 | 0.1 | 1.6 | 35 | 53 | 0.2 | 0.1 | 1.5 | 65 |
|  |  |  |  |  | 54 | 0.2 | 0.1 | 2.8 | 69 | 54 | 0.1 | 0.1 | 2.3 | 47 |
|  |  |  |  |  | 55 | 0.2 | 0.0 | 5.3 | 56 | 55 | 0.3 | 0.3 | 1.0 | 46 |
|  |  |  |  |  | 56 | 0.2 | 0.1 | 1.2 | 86 | 56 | 0.1 | 0.0 | 2.6 | 69 |
|  |  |  |  |  | 57 | 0.3 | 0.0 | 17.0 | 21 | 57 | 0.1 | 0.0 | 13.6 | 38 |
|  |  |  |  |  | 58 | 0.1 | 0.0 | 11.8 | 62 | 58 | 0.1 | 0.0 | 10.4 | 19 |
|  |  |  |  |  | 59 | 0.1 | 0.1 | 1.2 | 58 | 59 | 0.1 | 0.0 | 8.3 | 66 |
|  |  |  |  |  | 60 | 0.1 | 0.1 | 1.2 | 0 | 60 | 0.1 | 0.0 | 16.2 | 30 |
|  |  |  |  |  | 61 | 0.1 | 0.1 | 1.4 | 64 | 61 | 0.0 | 0.0 | 9.0 | 29 |
|  |  |  |  |  | 62 | 0.1 | 0.0 | 3.8 | 89 | 62 | 0.1 | 0.0 | 2.6 | 66 |
|  |  |  |  |  | 63 | 0.1 | 0.0 | 9.0 | 6 | 63 | 0.0 | 0.0 | 4.1 | 55 |
|  |  |  |  |  | 64 | 0.2 | 0.1 | 3.2 | 50 | 64 | 0.0 | 0.0 | 7.2 | 30 |
|  |  |  |  |  | 65 | 0.1 | 0.1 | 1.2 | 64 | 65 | 0.1 | 0.0 | 8.7 | 1 |
|  |  |  |  |  | 66 | 0.2 | 0.1 | 1.9 | 86 | 66 | 0.0 | 0.0 | 3.9 | 38 |
|  |  |  |  |  | 67 | 0.5 | 0.2 | 3.0 | 37 | 67 | 0.2 | 0.1 | 2.4 | 62 |
|  |  |  |  |  | 68 | 0.1 | 0.1 | 1.3 | 83 | 68 | 0.2 | 0.1 | 1.1 | 73 |
|  |  |  |  |  | 69 | 0.1 | 0.0 | 2.8 | 39 | 69 | 0.0 | 0.0 | 8.7 | 24 |
|  |  |  |  |  | 70 | 0.1 | 0.0 | 2.2 | 18 | 70 | 0.1 | 0.0 | 10.8 | 3 |
|  |  |  |  |  | 71 | 0.1 | 0.1 | 1.9 | 28 | 71 | 0.1 | 0.0 | 18.7 | 37 |
|  |  |  |  |  | 72 | 0.1 | 0.1 | 2.2 | 35 | 72 | 0.0 | 0.0 | 10.3 | 24 |
|  |  |  |  |  | 73 | 0.5 | 0.1 | 6.9 | 16 | 73 | 0.0 | 0.0 | 2.7 | 6 |
|  |  |  |  |  | 74 | 0.3 | 0.0 | 8.9 | 44 | 74 | 0.1 | 0.1 | 1.2 | 20 |
|  |  |  |  |  | 75 | 0.2 | 0.1 | 2.4 | 9 | 75 | 0.4 | 0.1 | 6.3 | 8 |
|  |  |  |  |  | 76 | 0.4 | 0.1 | 4.6 | 9 | 76 | 0.2 | 0.1 | 2.5 | 21 |
|  |  |  |  |  | 77 | 0.2 | 0.1 | 4.1 | 34 | 77 | 0.2 | 0.0 | 3.9 | 20 |
|  |  |  |  |  | 78 | 0.4 | 0.1 | 3.3 | 10 | 78 | 0.5 | 0.2 | 2.4 | 69 |
|  |  |  |  |  | 79 | 0.5 | 0.0 | 31.6 | 6 | 79 | 0.1 | 0.1 | 2.3 | 58 |
|  |  |  |  |  | 80 | 0.1 | 0.0 | 7.9 | 22 | 80 | 0.0 | 0.0 | 8.3 | 2 |
|  |  |  |  |  | 81 | 0.1 | 0.0 | 6.7 | 2 | 81 | 0.0 | 0.0 | 15.0 | 9 |
|  |  |  |  |  | 82 | 0.1 | 0.0 | 12.3 | 8 | 82 | 0.0 | 0.0 | 8.6 | 14 |
|  |  |  |  |  | 83 | 0.1 | 0.0 | 9.0 | 15 | 83 | 0.0 | 0.0 | 9.8 | 2 |
|  |  |  |  |  | 84 | 0.1 | 0.0 | 14.3 | 4 | 84 | 0.0 | 0.0 | 8.2 | 40 |
|  |  |  |  |  | 85 | 0.1 | 0.0 | 6.9 | 23 | 85 | 0.2 | 0.1 | 1.7 | 5 |
|  |  |  |  |  | 86 | 0.2 | 0.2 | 1.0 | 21 | 86 | 0.1 | 0.0 | 20.0 | 21 |
|  |  |  |  |  | 87 | 0.2 | 0.1 | 1.9 | 24 | 87 | 0.0 | 0.0 | 2.4 | 68 |
|  |  |  |  |  | 88 | 0.2 | 0.1 | 1.3 | 85 | 88 | 0.0 | 0.0 | 1.6 | 17 |
|  |  |  |  |  | 89 | 0.1 | 0.0 | 4.4 | 87 |  |  |  |  |  |
|  |  |  |  |  | 90 | 0.1 | 0.0 | 3.3 | 80 |  |  |  |  |  |
|  |  |  |  |  | 91 | 0.1 | 0.0 | 1.8 | 30 |  |  |  |  |  |
|  |  |  |  |  | 92 | 0.3 | 0.1 | 2.5 | 64 |  |  |  |  |  |

Supplementary Figure S1

Figure S1 shows the compositional variability of biotites in the proximal samples (clastic material, single clast) in a ternary AFM diagram determined by EMPA.

*
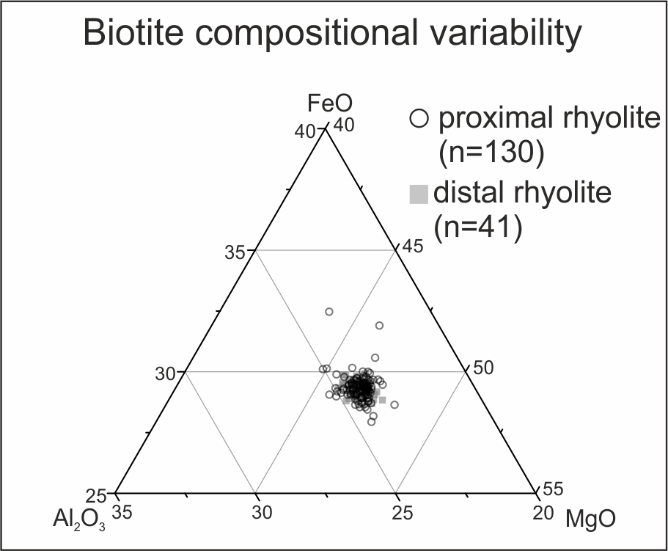
*

1. **Glass geochemistry of the rhyolite**

The spatial variation of major element chemistry was measured for the groundmass glass of the proximal and distal rhyolites by electron microprobe microanalysis of linear transects perpendicular to the margin. We used a defocussed 10 μm beam, with a 15 kV accelerating voltage and 5 nA beam current. We discarded measurements taken close to textural features, such as vesicles, and we measured alkali elements first (Na & K), to minimize artefacts associated with volatilisation of these elements by the electron beam. Calculated total oxide abundances are 98.5-101.5 wt.%, qualitatively suggesting that artefacts associated with excitation volumes exceeding vesicle wall thicknesses need not be considered.

The glass in the distal rhyolite is chemically homogeneous (within analytical error) on the millimetre scale (Fig. S2). In the proximal rhyolite, beyond 1 mm from the conduit margin, we note a similar chemical homogeneity in the glass phase of both the clastic and single clast samples. In the first millimetre of the conduit margin, however, the concentrations of Na_2_O and K_2_O are subtly depleted and enriched, respectively (Fig. S2). This trend is particularly clear when Na_2_O and K_2_O are normalised to a relatively less mobile element, such as SiO_2_ (analytical error of 0.52 wt.%; Fig. S2). Given the relatively low absolute abundances of Na_2_O and K_2_O and an average analytical error of 0.12 wt.% for these elements, this trend is subtle. Nonetheless, the data highlight a 1 mm thick, chemically distinct zone coincident with the observed physical modification at the conduit margin. Taken together, these chemical and textural signatures appear to underline the interaction of the wall rock with magma during eruption.

Supplementary Figure S2

Oxide distribution in the rhyolitic glass of the proximal samples as a function of distance from the marginal contact with the AD 1886 basalt eruption normalised to the abundance of SiO_2_. Normalised abundance of Na_2_O and K_2_O are shown as a moving average (dashed and dotted black curves; moving window: n=10). The grey shaded region marks +/- 1σ of the distal rhyolite; +/- 1σ of the clastic and the single clast sample have a similar extent but are left out for clarity. *Inset:* the raw data used in the averaging calculation. In all cases the mean and range of n=49 distal rhyolite measurements are shown as a grey line and a shaded region, respectively. Note the relative depletion of the Na_2_O ratio and enrichment of the K_2_O ratio close to the margin.


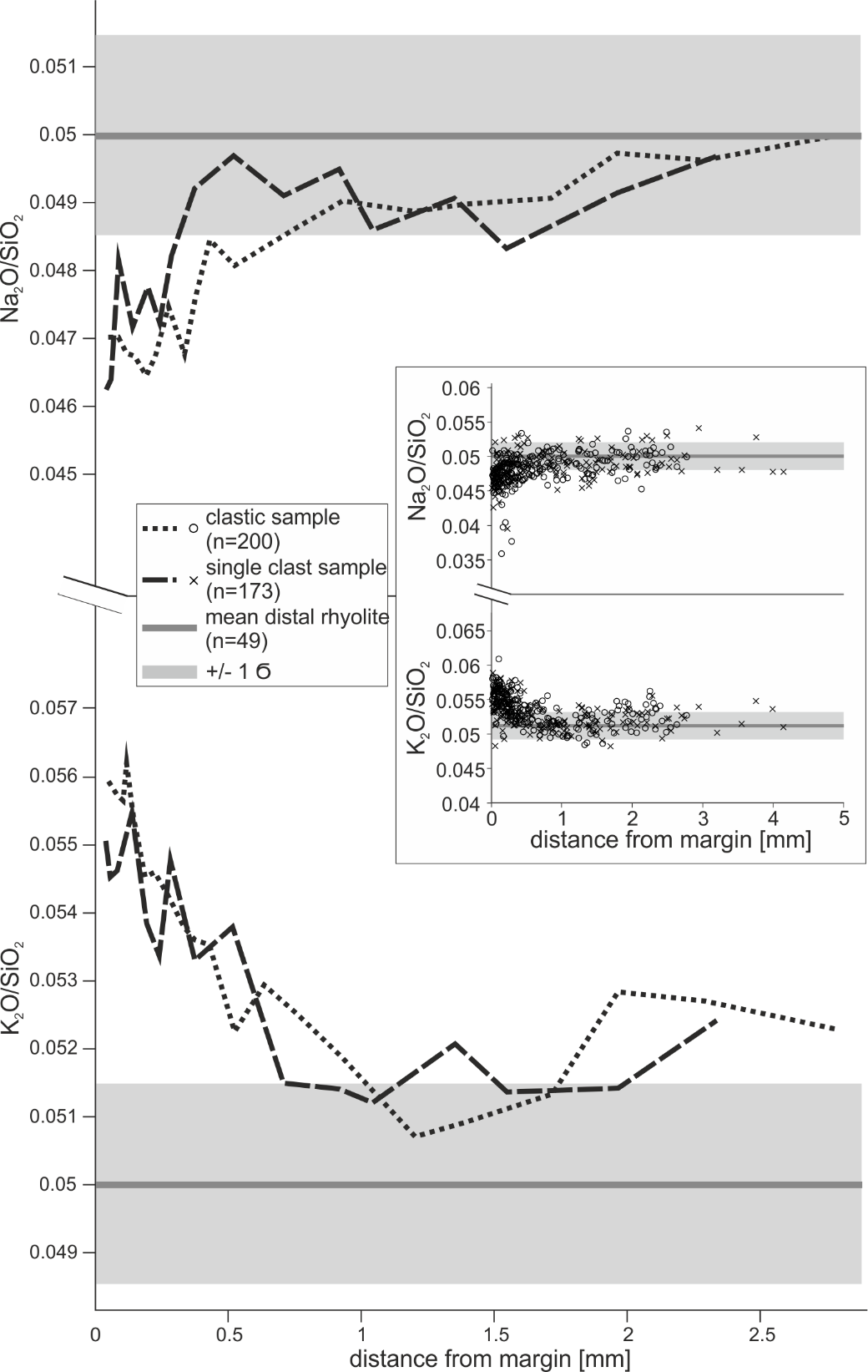


1. **Thermal diffusivity of rhyolites**

The nomenclature here used is fully explained in the main text. Bagdassarov and Dingwell (1994) propose that $D=9.14\cdot{10}^{-7}-1.4\cdot{10}^{-9}T+1.9\cdot{10}^{-12}T^{2}$. When this function is fit to Eq. 2 (main text) in the temperature range 450-850 °C, we find $D_{0}=3.66\cdot{10}^{-7}$ m^2^.s^-1^ and $\alpha=1.24\cdot{10}^{-3}$ K^-1^.

Supplementary Figure S3

Calibration of thermal diffusivity over the temperature range.


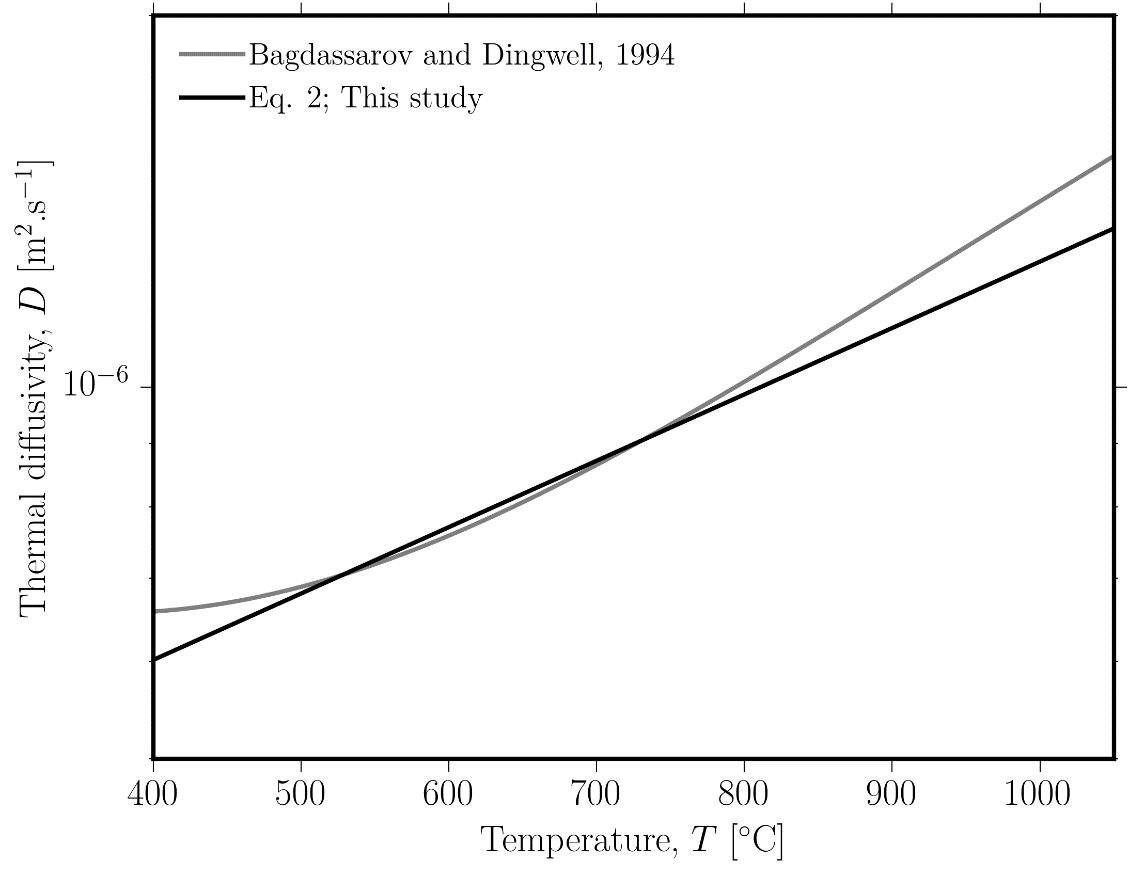

Supplement: Supplementary file 1 — (DOCX 387 kb) [file 445_2016_1006_MOESM1_ESM.docx]
